# Effect of Chemical Structure on the Performance of Sulfonated Poly(aryl ether sulfone) Composite Nanofiltration Membranes

**DOI:** 10.3390/membranes9010006

**Published:** 2019-01-02

**Authors:** Shouhai Zhang, Shanshan Guan, Chengde Liu, Zhenlin Wang, Danhui Wang, Xigao Jian

**Affiliations:** State Key Laboratory of Fine Chemicals, Liaoning High Performance Polymer Engineering Research Center, College of Chemical Engineering, Dalian University of Technology, Dalian 116024, China; guanshanshan.86@163.com (S.G.); liucd@dlut.edu.cn (C.L.); wzl1503@163.com (Z.W.); wangdh9300@mail.dlut.edu.cn (D.W.); jian4616@dlut.edu.cn (X.J.)

**Keywords:** sulfonated poly(aryl ether sulfone), phthalazinone, structure, composite membranes, nanofiltration

## Abstract

This paper discusses the effect of the chemical structure of sulfonated poly(aryl ether sulfone) on the performance of composite nanofiltration membranes. The composite nanofiltration membranes were fabricated by coating sulfonated poly(aryl ether sulfone) solution onto the top surface of poly(phthalazinone ether sulfone ketone) support membranes. Three kinds of sulfonated poly(aryl ether sulfone)s with different amounts of phthalazinone moieties, namely, sulfonated poly(phthalazinone ether sulfone) (SPPES), sulfonated poly(phthalazinone biphenyl ether sulfone) (SPPBES), and sulfonated poly(phthalazinone hydroquinone ether sulfone)s (SPPHES), were used as coating materials. The solvents used in preparing the coating solution were investigated and optimized. The separation properties, thermal stability, and chlorine resistance of composite membranes were determined. The structures and morphologies of membranes were characterized with FTIR and SEM, respectively. The membrane prepared from SPPES with more phthalazinone moiety groups showed high water flux and salt rejection. The salt rejection of composite membranes followed the order SPPES > SPPHES > SPPBES. The rejection of the three composite membranes decreased slightly with the solution temperature rising from 20 to 90 °C, while the composite membrane with SPPES as the active layer showed a higher increase in flux than others. The results indicate that SPPES composite membranes show better thermal stability than others.

## 1. Introduction

Access to secure, sustainable sources of fresh water is one of the urgent needs in this century [1,2]. Membrane separation technology, as a method for wastewater treatment, is considered as an economical and environmentally friendly process. The technology has attracted increasingly more attention because of its low energy consumption, more competitive operating cost, and separation without phase change. Desalination by means of membranes appears to be an environmentally low-impact and energy-efficient route to produce fresh water [3,4,5,6].

As one of the membrane separation processes, nanofiltration (NF) can be applied in various industrial fields because it can remove multivalent salts and organic solutes with low molecular weight [7,8,9,10,11]. NF membranes show lower rejection ability against monovalent metal ions than the multivalent ones. NF also has some advantages, such as low maintenance and operation cost, high rejection against multivalent metal ions, high flux, and low operational pressure [12,13,14,15,16]. Most NF membranes are composite membranes consisting of a porous substrate and a top-separating layer. The chemistry and performance of the two parts of composite membranes can be independently optimized to maximize the membrane performance [17]. The top-separating layer plays an important role in the selectivity and permeability of composite membranes, and it is usually prepared from charged polymer materials via different methods such as dip-coating, interfacial polymerization, and so on [18,19,20,21]. Suffering from the limitation of thermal stability of common polymers, most polymer composite membranes can only be applied below 50 °C. To prepare thermally stable membranes, membrane materials with high thermal resistance should be used.

**Figure 1:**
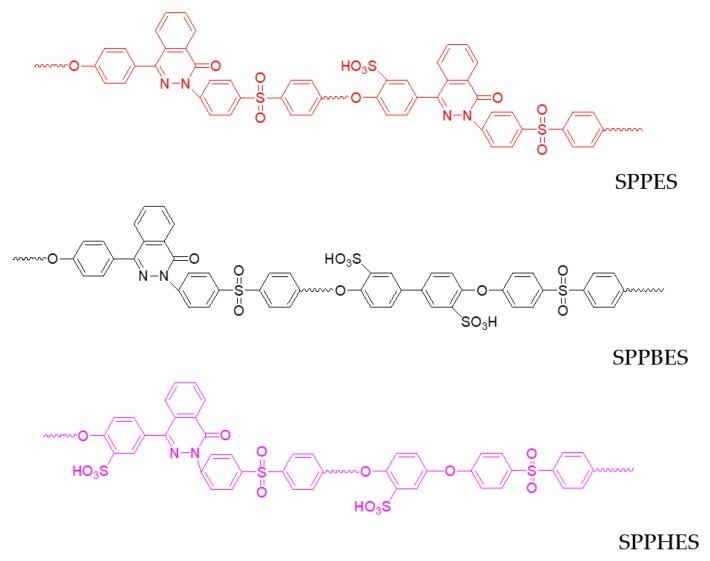
The chemical structure of three sulfonated poly(aryl ether sulfone)s: sulfonated poly(phthalazinone ether sulfone) (SPPES), sulfonated poly(phthalazinone biphenyl ether sulfone) (SPPBES), and sulfonated poly(phthalazinone hydroquinone ether sulfone)s (SPPHES).

A kind of poly(aryl ether)s containing phthalazinone moiety groups, including poly(phthalazinone ether sulfone) (PPES), poly(phthalazinone ether sulfone ketone)s (PPESK), poly(phthalazinone biphenyl ether sulfone)s (PPBES), and poly(phthalazinone hydroquinone ether sulfone)s (PPHES), were synthesized from 4-(4-hydroxyphenyl)-2,3-phthalazin-1-one (DHPZ) [22,23]. Sulfonated poly(aryl ether sulfone), such as SPPES, SPPBES, and SPPHES, were prepared from PPES, PPBES, and PPHES, respectively [24,25,26]. The chemical structure of SPPES, SPPBES, SPPHES are shown in Figure 1. These polymers exhibit outstanding thermal stabilities and excellent comprehensive properties and are considered as promising membrane materials. In our previous work, composite nanofiltration membranes were fabricated from sulfonated PPESK and SPPBES [27,28], and the effects of coating condition on the membrane performance were investigated. The primary properties of nanofiltration membranes are salt retention and water permeability, which depend on the polymer structure and properties of the top-separating layer [29,30]. However, a comparison and analysis of the relationship between the polymer structure and sulfonated poly(aryl ether sulfone) membrane performance has not yet been made.

In this work, a systematic study on sulfonated poly(aryl ether sulfone) nanofiltration membranes was performed to demonstrate the effect of chemical structure on membrane performance. The composite membranes were fabricated from sulfonated poly(aryl ether sulfone)s with different amounts of phthalazinone moieties and a similar degree of sulfonation. Scanning electron microscopy (SEM) and attenuated total reflectance infrared spectroscopy were used to characterize the structures and morphologies of the membranes. Separation properties, chlorine resistance, and thermal stability of the membranes prepared from different sulfonated poly(aryl ether sulfone)s were investigated.

## 2. Experiment

### 2.1. Materials and Instrument

PPESK was provided by Dalian New Polymer Co. (Dalian, China). SPPES, SPPBES, and SPPHES were synthesized according to the method previously reported [24,25,26]. SPPBES was obtained from the sulfonation of PPBES synthesized with the monomer ratio of DHPZ to biphenol (BP) of 6:4, and SPPHES was obtained from the sulfonation of PPHES synthesized with the monomer ratio of DHPZ to hydroquinone (HQ) of 4:6. The degree of sulfonation (DS) for each sulfonated poly(aryl ether sulfone) was in the range of 0.82–0.87. Ion exchange capacities of SPPES, SPPBES, and SPPHES were 1.67 mmol/g, 1.70 mmol/g, and 1.81 mmol/g, respectively. Ethanol, ethylene glycol monomethyl ether (EGME), 1,4-dioxane (DO), and acetone were all analytical grade and used directly. Na_2_SO_4_, MgSO_4_, NaCl, and MgCl_2_ were used to characterize the separation properties of membranes as solutes. A DDS-11A electrical conductivity instrument (Shanghai Leici Instrument, Shanghai, China) was used to determine the salt concentrations. A flat-sheet dead-end membrane cell with an effective volume of 550 mL was used to evaluate the performance of the composite membrane.

### 2.2. Solubilities of the Polymers

An amount of 0.01 g dry polymers (PPESK, SPPES, SPPBES, SPPHES) were immersed into 1 mL solvents at room temperature for 2 h, and the solubility of polymers was then observed.

### 2.3. Membrane Preparation

Composite membranes were fabricated following the same procedure as described in our previous work [28]. A 2 wt.% solution of sulfonated poly(aryl ether sulfone) in different solvent systems was prepared and filtered. PPESK ultrafiltration membrane with molecular weight cut-off of 10,000 Da was used as a substrate. The PPESK membrane was tapped on glass, and the sulfonated copoly(aryl ether sulfone) solution was then dropped on its surface. After the excess solution at the membrane surface was removed by holding the membrane vertically, the membrane was cured at 90 °C for 30 min. The resulting sulfonated poly(aryl ether sulfone) composite membrane was stored in deionized (DI) water before test.

### 2.4. Morphology and Structure

The morphologies of composite membranes were observed with SEM. The dried membrane samples were immersed into liquid nitrogen and fractured. After the samples were sputtered with gold, they were transferred to the SEM (QUANTA 450, FEI Company, Hillsboro, OR, USA) and measured.

Fourier transform infrared (FTIR) spectroscopy of PPESK and sulfonated poly(aryl ether sulfone) membranes were collected with a Nicolet-20DXB spectrometer (Nicolet Instrument Corporation, Madison, WI, USA) using attenuated total reflectance technique.

### 2.5. Water Flux and Salt Rejection

The performance of the resulting composite membranes was measured using a dead-end filtration set-up. First, membranes were prepressured at 1.2 MPa for half an hour with DI water. After that, flux and salt rejection were determined with 1.0 g/L salt aqueous solution under 1.0 MPa at ambient temperature. The flux was obtained by the following equation:

*F* = *Q*/(*At*)

where *F* is the permeate flux (L/(m^2^·h)), *A* is the effective area of the membrane (m^2^), *Q* is the volume of permeate solution (L), and *t* is the time (h).

The salt rejection (*R*) was calculated with following equation:

*R* = (1 − *C_p_*/*C_f_*) × 100%

where *C_p_* and *C_f_* are the salt concentrations in permeate and feed solution, respectively. The salt concentrations were obtained by determining the electrical conductance with a DDS-11A conductance meter (Shanghai Leici Instrument Co., Ltd., Shanghai, China). All of the test processes were repeated three times, and the average values were obtained.

### 2.6. Thermal Stability and Chlorine Resistance

To investigate the thermal stability of sulfonated poly(aryl ether sulfone) composite membranes, the separation properties of the membranes were tested with 1.0 g/L salt aqueous solution under a pressure of 1.0 MPa at the elevated operating temperature from 20 to 90 °C. In each step, the operating temperature was kept constant for at least 30 min.

To investigate chlorine resistance of membranes, sulfonated poly(aryl ether sulfone) composite membranes were immersed into 0.2 g/L sodium hypochlorite solution for 10 days. The membrane sample was taken out and washed with DI water at an interval of 2 days, then the solution flux and rejection were determined with 1.0 g/L Na_2_SO_4_ as feed solution under 1.0 MPa at room temperature.

## 3. Results and Discussion

### 3.1. Effect of Solvents Used in the Coating Solutions

For preparation of composite membranes by dip-coating method, the solvents used in the coating solution should dissolve the coating materials and not damage the substrate membranes. The solubility of polymers in solvents can be evaluated by solubility parameters. Bagley et al. [31] reported that the effect of polar solubility parameter (*δ_d_*) and dispersion solubility parameter (*δ_p_*) was very similar, but the effect of hydrogen bonding solubility parameter (*δ_h_*) was completely different. Therefore, the introduced volume solubility parameter (*δ_v_*) is defined as follows:

(1) $$\delta_{v}=\sqrt{\delta_{d}^{2}+\delta_{p}^{2}}$$

(2) $$\Delta \delta =\sqrt{{\left(\delta_{v1}-\delta_{v2}\right)}^{2}+{\left(\delta_{h1}-\delta_{h2}\right)}^{2}}$$

where *δ_d_*, *δ_p_*, and *δ_h_* refer to dispersion, polar, and hydrogen bonding components of the solubility parameter, respectively; subscripts 1 and 2 refer to polymer and solvent, respectively; and Δ*δ* is the difference in solubility parameter between the polymer and the solvent. The smaller the Δ*δ* value, the better is the solubility of the polymer in the solvent. Generally speaking, polymer can be dissolved in the solvent when Δ*δ* value is less than 5 MPa^1/2^ [32]. The solubility parameter of PPESK and sulfonated poly(aryl ether sulfone)s were calculated according to our previous work [33] and are shown in Table 1. Sulfonated poly(aryl ether sulfone) showed higher *δ_h_* than PPESK, while the *δ_v_* was comparable. Based on Equations (1) and (2), the difference in solubility parameter between solvent and polymer were obtained and are listed in Table 2. For the solvent systems, including EGME, EGME + acetone (5:1), EGME + DO (4:1), and EGME + ethanol (4:1), the Δ*δ* values between solvent and sulfonated poly(aryl ether sulfone) were less than or close to 5 MPa^1/2^. For the same solvent, the Δ*δ* value showed the order SPPHES < SPPBES ≤ SPPES. However, the difference in Δ*δ* value was no more than 0.6 MPa^1/2^. This indicated that there was no significant change on the solubility of sulfonated poly(aryl ether sulfone). The Δ*δ* values between solvent and PPESK were more than 7.3 MPa^1/2^, indicating that PPESK showed poor solubility in these solvents. The results were confirmed by the solubility test. From Table 3, it can be seen that SPPES, SPPBES, SPPHES were soluble in the four solvents, and PPESK was insoluble in them. Therefore, these solvent systems could be used as solvents to prepare the coating solution.

Hamza et al. [34] reported that solvents used in preparing the coating solution had a great effect on the performance of composite membranes. To investigate the effect of solvents on the membrane performance, EGME, EGME + acetone (5:1), EGME + DO (4:1), and EGME + ethanol (4:1) were used as the coating solvents to prepare sulfonated poly(aryl ether sulfone) composite membranes. The performance of composite membranes for a 1.0 g/L Na_2_SO_4_ feed solution was measured, and the results are shown in Table 4. SPPES composite membrane prepared from EGME + acetone (5:1) as the solvent had the highest Na_2_SO_4_ rejection (90%), SPPBES composite membrane fabricated from EGME as the solvent had the highest Na_2_SO_4_ rejection (86%), and SPPHES composite membrane prepared from EGME + DO (4:1) as the solvent had the highest Na_2_SO_4_ rejection (87%), However, composite membranes prepared from these solvents showed relatively low fluxes. With EGME + ethanol (4:1) as the solvent, the Na_2_SO_4_ rejection of SPPES membrane was 88%, and the flux was 55 L/(m^2^·h), while the SPPHES membrane showed 85% Na_2_SO_4_ rejection. With sulfonated poly(aryl ether sulfone) composite membranes prepared with EGME + ethanol (4:1) as the solvent, the composite membranes showed high rejection and flux. The rejection of composite membranes ranged from 81% to 88%, and the flux of composite membranes was in the range of 45–55 L/(m^2^·h).

### 3.2. Separation Performance of Sulfonated Poly(aryl ether sulfone) Composite Nanofiltration Membrane

EGME + ethanol (4:1) was selected as the solvent for preparing sulfonated poly(aryl ether sulfone) solutions. Composite membranes were fabricated from these solutions. The membrane performance for 1.0 g/L of different salt solutions was tested under 1.0 MPa pressure. The test results are shown in Table 5. The average pure water flux (PWF) was 70 L/(m^2^·h), 69 L/(m^2^·h), and 60 L/(m^2^·h) for the SPPES, SPPBES, and SPPHES membranes, respectively. In addition, it was observed that SPPES composite membrane showed high salt rejection as well as high pure water flux. It is known that the permselectivity of composite membranes mainly depends on the active layer [35]. Compared with SPPBES and SPPHES, SPPES containing more phthalazinone moiety groups had more free volume and thus enhanced the permeability of SPPES composite membranes. Experimental results revealed that the salt rejection of composite membranes followed the sequence SPPES > SPPHES > SPPBES. The salt rejection decreased in the order Na_2_SO_4_ > MgSO_4_ > NaCl > MgCl_2_ for composite membranes thus prepared. This is because the rejection of composite membranes is mainly influenced by Donnan effect. The sulfonic acid groups on the surface of composite membranes are negatively charged. The negatively charged groups show higher exclusion effect of divalent anions than monovalent ones and higher absorption of divalent cations than monovalent ones. Therefore, the rejection for divalent anion is higher than for monovalent anion, while the rejection for cations is in the reverse order. This sequence agrees with the Donnan characteristic of salt rejection for a negatively charged membrane [18].

### 3.3. Performance of Composite Membranes with Different Selective Layers at Increasing Solution Temperature

To investigate the thermal stability of membranes, the main method is to evaluate their separation properties under various temperatures of feed solutions. Solute rejection and flux of polymer membranes is related to the mobility of the macromolecular chain. Molecular chains of polymers become more flexible and their shapes became easier to change as solution temperature rises. The molecular chain is more sensitive to temperature, and the membrane pore is more likely to change under hydraulic pressure at an elevated solution temperature [35].

**Figure 2:**
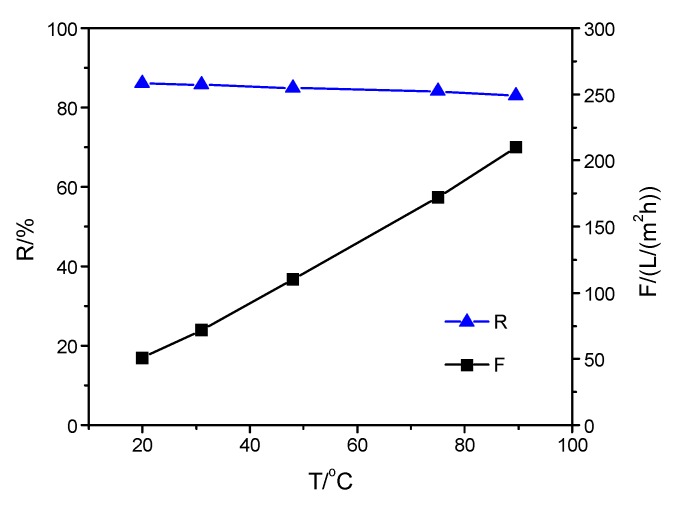
Influence of operating temperature on the properties of SPPES composite membrane.

**Figure 3:**
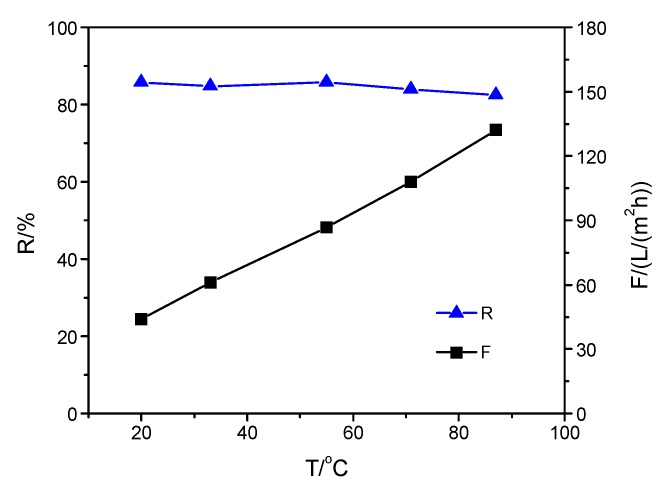
Influence of operating temperature on the properties of SPPBES composite membrane.

**Figure 4:**
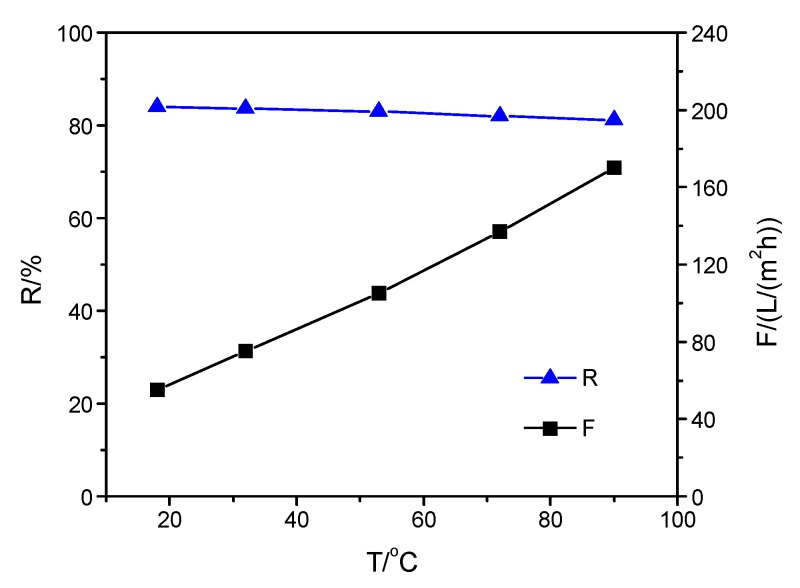
Influence of operating temperature on the properties of SPPHES composite membrane.

To investigate the effect of the top-separating layer structure on the thermal stability of the composite membrane, the separation properties of the composite membranes were measured when the operating temperature was increased from 20 to 90 °C. The effect of operating temperature on the properties of the three membranes is shown in Figure 2, Figure 3 and Figure 4. As shown in Figure 2, there was a slight change in Na_2_SO_4_ rejection of SPPES membrane from 86.0% to 82.9% when the solution temperature increased from 20 to 90 °C, while the flux increased 4.1 times. In Figure 3 and Figure 4, a similar tendency for rejection of membranes with the same operating condition can be observed, and the difference of the rejection of each membrane was less than 3.2%. With the solution temperature rising from 20 to 90 °C, the flux of SPPBES membrane and SPPHES composite membrane increased 3 times and 3.1 times, respectively. This indicates that the composite membranes with different sulfonated poly(aryl ether sulfone)s as the active layer show good thermal stability. As the solution temperature increased, the composite membrane with SPPES as active layer showed a higher increase in flux than the others. This was mainly because sulfonated poly(aryl ether sulfone)s with similar degree of sulfonation (SPPES, SPPBES, and SPPHES) were prepared from PPES, PPBES, and PPHES. The glass transition temperature of PPES, PPBES, and PPHES is 305, 273, and 233 °C, respectively [22,23]. Due to containing the most phthalazinone moiety groups in the polymer chains, PPES showed the highest thermal stability among them. Although the glass transition temperature of sulfonated poly(aryl ether sulfone)s could not be determined due to the decomposition of sulfonic acid groups over 250 °C, it can be concluded that SPPES should have the highest thermal stability among the three sulfonated polymers. Compared with SPPBES and SPPHES, the motion of the molecular chain of SPPES and the structural change of composite membranes were more limited at a high temperature, while the viscosity of water greatly decreased with an increase in solution temperature, leading to the increase in flux. SPPES composite membrane showed better thermal stability than SPPBES and SPPHES membranes.

### 3.4. Chlorine Resistance of Sulfonated Poly(aryl ether sulfone) Composite Membranes

**Figure 5:**
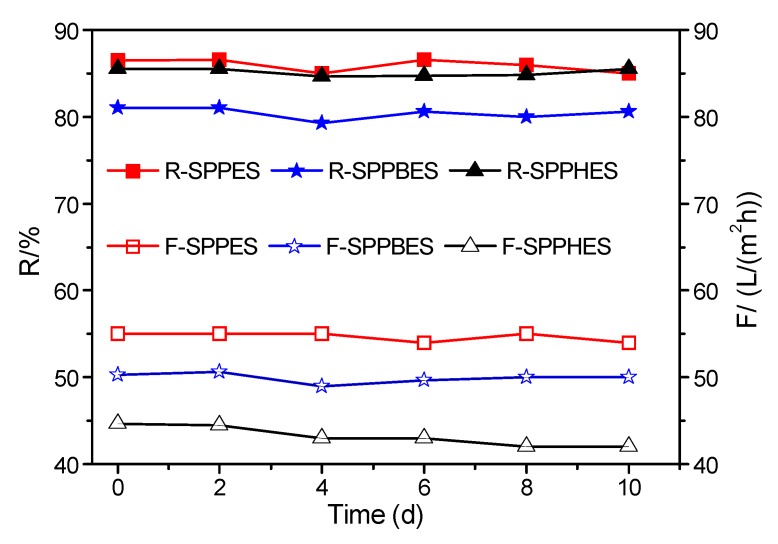
Chlorine resistance of composite membrane.

Chlorine resistance of membranes is a very important parameter for nanofiltration applications. To investigate chlorine resistance of membranes, the stability of sulfonated poly(aryl ether sulfone) composite membranes in 0.2 g/L sodium hypochlorite solution was evaluated. The results are shown in Figure 5. After being immersed in sodium hypochlorite solution for 10 days, the solution flux of SPPES, SPPBES, and SPPHES composite membranes decreased 1.0, 1.3, and 2.7 L/(m^2^·h), and the rejection of SPPES, SPPBES, and SPPHES membranes decreased 1.5%, 1.7%, and 0.9%, respectively. There was no significant difference in the chlorine resistance of the prepared composite membranes. The results indicate that all three sulfonated poly(aryl ether sulfone) composite membranes show good chlorine resistance and are superior to polyamide commercial composite membranes.

Although these sulfonated poly(aryl ether sulfone) composite membranes show high thermal stability and chlorine resistance, they show relative lower flux than those of polyamide commercial composite membranes [20,21]. Further work is under way to improve the flux of sulfonated poly(aryl ether sulfone) composite membranes.

### 3.5. Fourier Transform Infrared Spectroscopy of Sulfonated Poly(aryl ether sulfone) Composite Membranes

**Figure 6:**
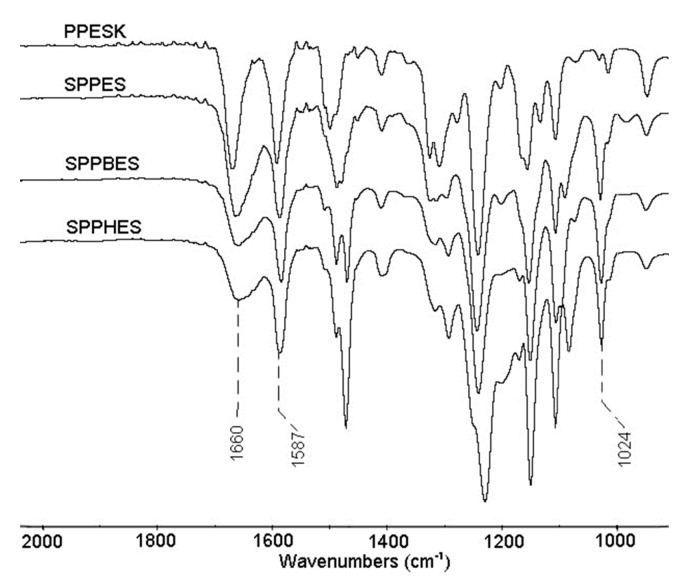
FTIR spectra of PPESK membrane and SPPES, SPPBES, and SPPHES composite membranes.

Figure 6 illustrates the FTIR spectra of PPESK substrate and SPPES, SPPBES, and SPPHES composite membranes between 800 and 2000 cm^−1^. As shown in Figure 6, the peak exhibited at 1660 cm^−1^ confirmed the appearance of aromatic carbonyl C=O in PPESK, SPPES, SPPBES, and SPPHES. The peak at 1587 cm^−1^ can be considered as a C=C in the benzene skeleton. The strength of the peak at 1660 cm^−1^ decreased in the order of PPESK = SPPES > SPPBES > SPPHES. This was mainly because the content of phthalazinone moiety groups in these polymers deceased in the same order. A new absorption at 1024 cm^−1^ appeared in the spectra of SPPES, SPPBES, and SPPHES composite membranes, while it was absent in the spectrum of PPESK. The peak can be attributed to the characteristic absorption of O=S=O in sulfonic acid groups [28]. The results indicate that composite nanofiltration membranes can be successfully fabricated by coating sulfonated poly(aryl ether sulfone) on PPESK ultrafiltration support membrane.

### 3.6. Morphological Structure of the Sulfonated Poly(aryl ether sulfone) Composite Membranes

**Figure 7:**
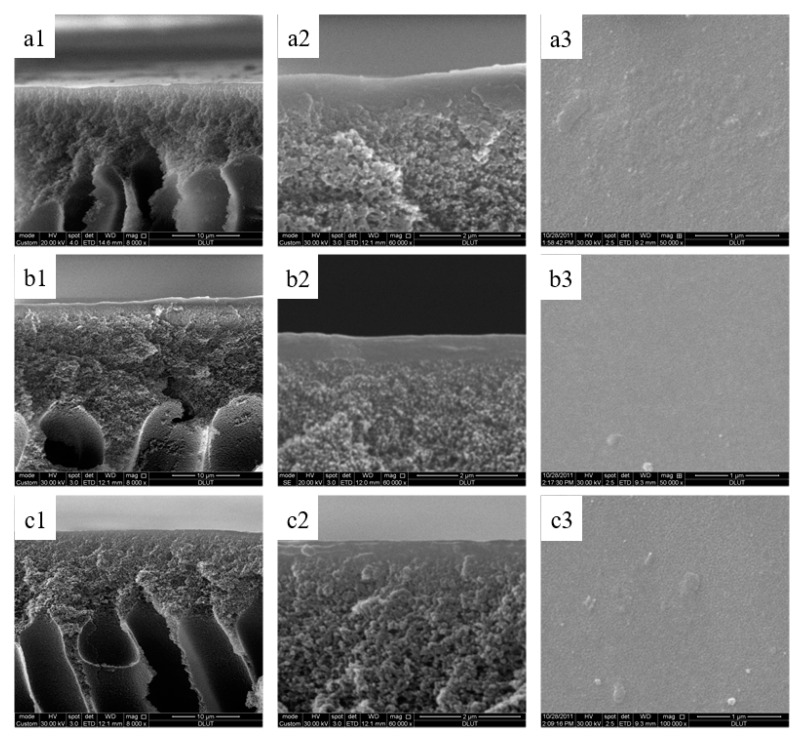
The morphologies of composite membrane. (**a1**,**b1**,**c1**) are the cross sections of SPPES, SPPBES, and SPPHES, respectively, at 8000×. (**a2**,**b2**,**c2**) are the cross sections of SPPES, SPPBES, and SPPHES, respectively, at 60,000×. (**a3**,**b3**,**c3**) are the surface of SPPES, SPPBES, and SPPHES, respectively.

SEM images of the cross section and surface of SPPES, SPPBES, and SPPHES composite membranes are shown in Figure 7. SPPES, SPPBES, and SPPHES composite nanofiltration membranes took on a composite structure, namely, a thin active layer appearing on the porous PPESK support membrane. The effective thickness of the skin layer of the composite membranes was approximately 0.5 μm. The top surface feature of the three composite membranes appeared to be dense and smooth. There was no obvious difference in the morphologies between SPPES, SPPBES, and SPPHES composite nanofiltration membranes. The morphologies of the membranes illustrated that the composite membranes with dense separating layer were successfully fabricated on the substrate.

## 4. Conclusions

Composite membranes were prepared using sulfonated copoly(aryl ether sulfone) with different amounts of phthalazinone moieties (SPPES, SPPBES, SPPHES) as the selective layer via the dip-coating method. Four solvent systems used in preparing the coating solution were investigated, and EGME + ethanol (4:1) was selected as the optimal solvent system. The rejection of composite membranes with different active layers followed the sequence SPPES > SPPHES > SPPBES. The results revealed that SPPES composite membranes exhibited higher water flux and salt rejection than SPPBES and SPPHES. Composite membranes showed nanofiltration characteristics. The salt rejection of the three composite membranes decreased in the order Na_2_SO_4_ > MgSO_4_ > NaCl > MgCl_2_. The rejection of the three composite membranes decreased slightly as the solution temperature was raised from 20 to 90 °C, while the SPPES composite membrane showed a higher increase in the flux than others. The results indicate that composite membranes show good thermal stability, and the SPPES membrane shows better separation properties and thermal stability than others. After being immersed in 0.2 g/L sodium hypochlorite solution for 10 days, the solution flux of composite membranes decreased less than 1.7%, indicating the good chlorine resistance of the three sulfonated poly(aryl ether sulfone) composite membranes. There was no obvious difference in the morphologies between SPPES, SPPBES, and SPPHES composite nanofiltration membranes.

## Figures and Tables

**Table 1:** Solubility parameters of poly(phthalazinone ether sulfone ketone)s (PPESK) and sulfonated poly(aryl ether sulfone)s.

Polymer	*δ_d_*/MPa^1/2^	*δ_p_*/MPa^1/2^	*δ_h_*/MPa^1/2^	*δ_v_*/MPa^1/2^
PPESK	20.4	5.7	7.8	21.2
SPPES (DS = 0.87)	20.0	7.3	11.9	21.3
SPPBES (DS = 0.85)	19.4	7.1	11.6	20.6
SPPHES (DS = 0.82)	19.1	7.8	12.1	20.6

**Table 2:** Comparison of solubility parameters between sulfonated poly(aryl ether sulfone)s, PPESK, and solvents.

Solvent	*δ_d_*/MPa^1/2^	*δ_p_*/MPa^1/2^	*δ_v_*/MPa^1/2^	*δ_h_*/MPa^1/2^	Δ*δ*/MPa^1/2^			
					SPPES	SPPBES	SPPHES	PPESK
EGME	16.2	9.2	18.6	16.4	5.2	5.2	4.7	9.0
EGME + acetone (5:1)	16.1	9.4	18.6	14.8	4.0	3.8	3.4	7.5
EGME + DO (4:1)	16.8	7.7	18.5	14.6	3.9	3.7	3.3	7.3
EGME + ethanol (4:1)	16.1	9.1	18.5	17.0	5.8	5.8	5.3	9.6

**Table 3:** Solubility of PPESK and sulfonated poly(aryl ether sulfone)s.

Solvent	PPESK	SPPES (0.87)	SPPBES (0.85)	SPPHES (0.82)
EGME	−	+	+	+
EGME + acetone (5:1)	−	+	+	+
EGME + DO (4:1)	−	+	+	+
EGME + ethanol (4:1)	−	+	+	+

Solubility: + soluble; − insoluble.

**Table 4:** Effect of solvents used in the coating solution.

Solvents	SPPES		SPPBES		SPPHES	
	*R*/%	*F*/(L/(m^2^·h))	*R*/%	*F*/(L/(m^2^·h))	*R*/%	*F*/(L/(m^2^·h))
EGME + acetone (5:1)	90	32	80	59	74	50
EGME + DO (4:1)	85	50	80	43	87	31
EGME + ethanol (4:1)	88	55	81	50	85	45
EGME	80	64	86	16	80	50

**Table 5:** Separation performance of sulfonated poly(aryl ether sulfone) composite membrane.

Membrane	PWF/(L/(m^2^·h))	*R*/%			
		Na_2_SO_4_	MgSO_4_	NaCl	MgCl_2_
SPPES	70	85	53	47	14
SPPBES	69	77	31	20	10
SPPHES	60	80	38	35	14
